# Enhanced drug specific CD8+ T cell response at the presence of HHV-6 in DRESS patient: a case report

**DOI:** 10.1186/2045-7022-4-S3-P86

**Published:** 2014-07-18

**Authors:** Hyun Seung Lee, Kyung-Hwan Lim, Ji Won Lee, Hye-Ryun Kang

**Affiliations:** 1 Seoul National University Medical Research Center, Institute of Allergy and Clinical Immunology, Korea, Republic of; 2 Seoul National University College of Medicine, Department of Internal Medicine, Korea, Republic of; 3 Seoul National University Hospital, Regional Pharmacovigilance Center, Korea, Republic of

## Background

Drug rash with eosinophilia and systemic symptoms (DRESS) syndrome is a potentially life-threatening multiorgan systemic reaction that includes skin rash, fever, lymphadenopathy, internal organ involvement, and leukocytosis with eosinophilia. Evidence for reactivation of human herpes virus (HHV) 6 has been seen in some DRESS patients. We present a case of allopurinol-induced DRESS syndrome and describe the immune reactions through the identification of proliferation of CD8-positive T lymphocytes against HHV-6 peptides pool (HHV6-U90) by flow cytometry.

## Case history

A 75-year-old man presented with a 10-day history of generalized rash accompanied by high fever, facial edema, hepatitis and azotemia. Prior to the appearance of the rash, the patient had been treated with allopurinol 200 mg/day due to gout for 20 days. Progressive rash, peripheral blood eosinophilia, multiple lymphadenopathy as well as a positive PCR test for HHV-6 were also present. The diagnosis of DRESS was made. The clinical symptoms and laboratory abnormalities were gradually recovered following administration of systemic corticosteroids (methylprednisolone 1 mg/kg).

## Result

Five years after the resolution of symptoms, lymphocyte transformation test was performed but significant lymphocyte proliferation was not observed with allopurinol stimulation itself in vitro. As a next step, CFSE T cell proliferation assays were conducted with the peripheral blood mononuclear cells of the allopurinol-induced DRESS patient and normal control. CD8+ cytotoxic proliferating T cells were not changed with stimulation of allopurinol in both DRESS patient and normal control. However, in DRESS patient, CD8+ cytotoxic proliferating T cell population was increased with stimulation of allopurinol and HHV6-U90 (33.9%¡æ41.2%).

## Conclusion

This result suggests that T cell proliferation assay with stimulation of both drug and HHV-6 could be a helpful method to detect the causative agent.

